# Gender determines the effect of atracurium priming technique in a randomized study

**DOI:** 10.12669/pjms.292.3163

**Published:** 2013-04

**Authors:** Liu Hui, Gu Lianbing, Zuo Yunxia

**Affiliations:** 1 Liu Hui, PhD, Department of Anesthesiology, West China Hospital, Sichuan University, Sichuan, 610041, China. Department of Anesthesiology, Jiangsu Cancer Hospital, Jiangsu, 210009, China.; 2 Gu Lianbing, MD,; 3 Zuo Yunxia, MD, Department of Anesthesiology, West China Hospital, Sichuan University, Sichuan, 610041, China. Department of Anesthesiology, Jiangsu Cancer Hospital, Jiangsu, 210009, China.

**Keywords:** Atracurium, Priming technique, Gender, Intubation time

## Abstract

***Objective:*** To evaluate the effect of priming atracurium over onset time and intubating time of general anesthesia between different genders.

***Methodology:*** Sixty-six male and sixty-four female patients, ASA I-II, aged 18-65 years, were randomly divided into four groups: group M_1_: male patients with saline priming; group M_2_: male patients with priming atracurium dose of 0.05 mg/kg; group F_1_: female patients with saline priming; group F_2_: female patients with priming atracurium dose of 0.05 mg/kg. General anesthesia was induced with midazolam(0.1 mg*kg^-1^) propofol(0.75 mg*kg^-1^), intubation dose of atracurium (0.5 mg*kg^-1^), fentanyl (3 μg*kg^-1^). The incidences of dizziness, diplopia, heavy eyelids and dyspnea were observed. Neuromuscular tension was quantified by using TOF-Guard neuromuscular monitor, and intubating time was defined as the duration from the infusion of intubation dose of atracurium to the time when T4/T1=0.

***Results: ***The intubating time of group F2 was shorter than that of group F1. There was no significant difference between group M1 and group M2. The incidences of dizziness, diplopia and heavy eyelids in group F2 were higher than those in group M2.

***Conclusion:*** Atracurium priming technique could shorten the intubation time of female patients, but not for male patients, and the gender plays a key role in affecting the clinical outcome of atracurium priming.

## INTRODUCTION

Atracurium is one of the non-depolarization neuromuscular blocking agents, and can be used safely for patients with malfunction in liver and kidney owing to its unique degradation of ester hydrolysis and the Hoffman reaction.^1^ Its onset time is slower than succinylcholine^2^, which limits the usage in rapid-sequence inducing of general anesthesia. Atracurium priming is one of the methods to shorten the onset time. Some studies have suggested that it was helpful to speed up the onset time. Atracurium priming with dose of 0.1mg/kg or 0.09mg/kg could shorten the onset time of atracurium.^3^^,^^4^ However some other researchers did not support this conclusion.^5^ Although the result is controversial, up to now, no studies have evaluated whether the gender is a key factor to affect the outcome of atracurium priming. So we aimed to investigate the different effect of priming atracurium between male and female patients.

## METHODOLOGY

This study was approved by hospital ethics committee (Jiangsu Cancer Hospital, 2011RA0028). One hundred thirty patients(sixty-six males and sixty-four females), aged from 18 to 65 years, ASA physical status I or II, 19< BMI (body mass index)<25 (Kg/m^2^), scheduled for elective surgery were entered into the study. Exclusive criteria were the presence of abnormal airway, diseases in cardiovascular and respiratory system, malfunction of neuromuscular, hepatic or renal system, and current drug administration known or suspected to interfere with neuromuscular transmission. All patients were randomized into four groups according to the method of random digits generated by SPSS software: group M_1_: thirty-four male patients, with saline priming; group M_2_: thirty-two male patients, with atracurium priming (0.05 mg/kg, 1/10 of intubating dose); group F_1_: thirty-two female patients, with saline priming; group F_2_: thirty-two female patients, with priming atracurium (0.05 mg/kg, 1/10 of intubating dose). All drugs were prepared by the nurse and the priming drug (either saline or atracurium) were labeled with the same name. The doctor who performed the general anesthesia also did not know which group the patient belonged to. And the patient also did not know which group he or she belonged to. Atracurium or saline priming was performed three minutes before induction of general anesthesia. After the priming dose was administrated, reactions of patients including dizziness, diplopia, heavy eyelids and dyspnea were observed. General anesthesia was induced with midazolam (0.1 mg/kg, speed was 5 mg/10seconds), propofol (0.75 mg/kg, 20 mg/10s), intubation dose of atracurium (0.5 mg/kg, 1mg/s), fentanyl (3 μg/kg, 50ug/10s). Neuromuscular tension was quantified by using TOF-Guard neuromuscular monitor (HXD-I, HuaXiang, China).The ulnar nerve was stimulated supramaximally at the wrist with square pulses of 0.2 milliseconds duration, delivered in a train-of-four (TOF) sequence at 2 Hz every 12 seconds. The TOF values were displayed and recorded. The TOF ratio (the amplitude of the fourth evoked response as a fraction of the first evoked response, T4/T1) was recorded continuously after the patients were sedated. After the intubation dose of atracurium was infused, the duration from the time of ending infusion of atracurium to the time of T4/T1=0 was recorded as tracheal intubation time.

The sample size was calculated assuming a power level of 0.90 at the significance of 0.05, and the means of intubating time in four groups with 135 seconds,166 s,152 s,183 s, the most deviation in groups with 48.4 s, on the basis of our preliminary test. As a result, 28 patients in each group were needed. In case of some patients with unanticipated difficult intubation, we enrolled 130 patients.

Data were analyzed with one-way ANOVA (multiple comparisons were analyzed with Tukey HSD) and chi square test according to different types of data, all of which were performed with the SPSS Software Package (Version 17.0). Differences were considered significantly at *P< *0.05, and all values were given as mean ± standard deviation (SD).

## RESULT

**Fig.1:**
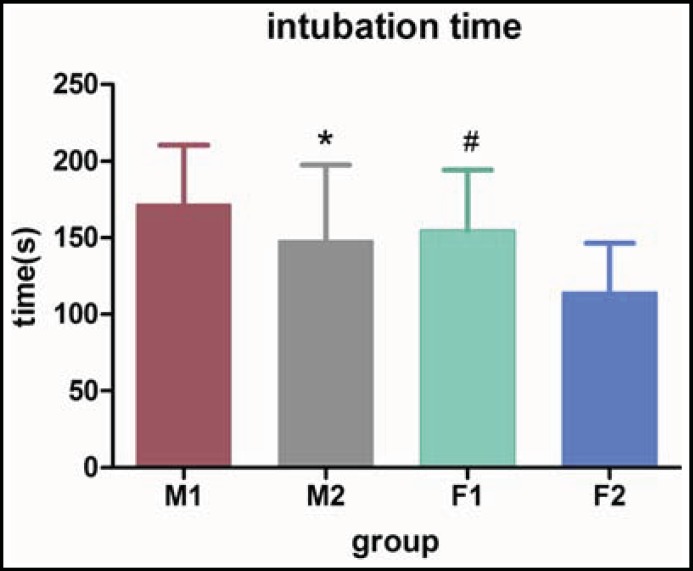
Intubation time of four groups The intubating time of group F2 is faster than the time of group F1(#, P=0.001), and also quicker than group M2 (*, P=0.008).

**Fig.2:**
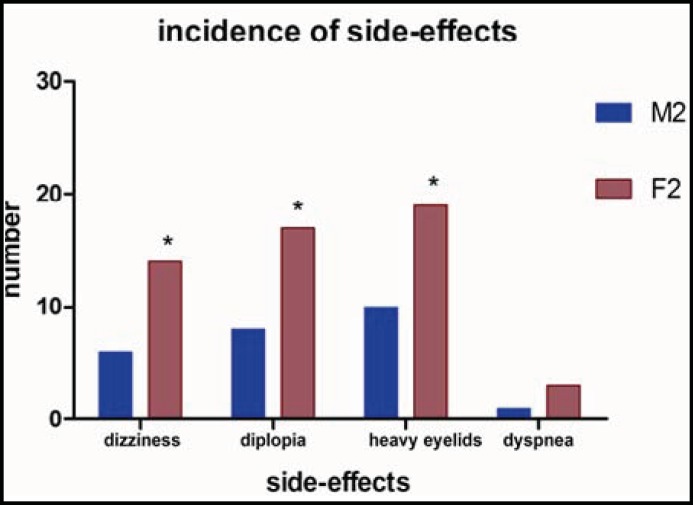
Incidences of side-effects between group M2 and group F2 The incidences of dizziness, diplopia and heavy eyelids of group F2 is higher than that of group M2 (*, p<0.05)

Six males and four females were finally ruled out for unanticipated difficulty in tracheal intubation. There were no significant differences between group M_1_ and group M_2_ in age, weight, height and BMI and no significant differences were found between group F_1_ and group F_2_ on age, weight, height and BMI (Table-I). There were no significant variation among four groups on age and BMI (Table-II). The tracheal intubation time of four groups were compared (Fig.1). Intubating time in group F2 was the least, (114.4±31.8 seconds, the 95% confidence interval for mean:102.6-126.3 s); intubating time of group F1 was 154.8±39.2 s (95% CI: 140.2-169.4 s); intubating time of group M2 was 148.3±49.2 s (95% CI: 129.9-166.7 s); intubating time of group M1 was 171.7±38.8 s (95% CI: 157.2-186.2 s). The side effects of group F2 and group M2 were observed, and the incidences in group F2 were 46.7%, 56.7%, 63.3%, 10.0%; the incidences in group M2 were 20.0%, 26.7%, 33.3%, 3.3% for dizziness, diplopia, heavy eyelids and dyspnea, respectively (Fig.2).

**Table-I:** Normal data in four groups (－ｘ±s).

*Group*	*Age (years)*	*Weight (kg)*	*Height (m)*	*BMI (kg/m* ^2^ *)*
M1	53.5±6.8	67.3±6.3	1.72±0.36	22.7±1.7
M2	52.8±7.3	66.7±6.8	1.71±0.31	22.8±1.9
F1	53.1±6.4	57.2±4.2	1.61±0.29	21.9±1.5
F2	52.1±6.4	58.2±4.9	1.60±0.37	22.6±1.5

Group M1 and M2: Age: P=0.703; weight: P=0.755; height: P=0.231; BMI: P=0.84

Group F1 and F2: Age: P=0.561; weight: P=0.43; height: P=0.236; BMI: P=0.103

**Table-II:** Normal data in four groups (±s).

*Group*	*Age (years)*	*BMI (kg/m* ^2^ *)*
M1	53.5±6.8	22.7±1.7
M2	52.8±7.3	22.8±1.9
F1	53.1±6.4	21.9±1.5
F2	52.1±6.4	22.6±1.5

Age: P=0.871; BMI: P=0.159

## DISCUSSION

Among the neuromuscular blocking agents, suxamethonium may be the fastest drug over onset time, as one kind of depolarization muscular relaxants, but the side effects of succinylcholine, such as hyperkalemia, intracranial hypertension, myalgias and others has limited its clinical use.^6^ So atracurium, one special non-depolarization neuromuscular blocking agent that is metabolized and eliminated independently on hepatic and renal function^1^, is regarded as an optimal candidate for inducing in general anesthesia. It is cheaper than cisatracurium and recuronium. However, the onset time of atracurium is slower than suxamethonium and some other non-depolarization muscular relaxants.^7^ So how to accelerate the onset time and to shorten the intubating time was the main problem in its clinical use in general anesthesia. Enlarging the dose of intubation or to increasing the infusion speed may increase the incidences of hypotension and tachycardia related to histamine release.^8^^-^^10^ The other way is to apply priming technique. Priming atracurium with 0.05mg/kg could shorten the intubating time of atracurium itself,^11^ although atracurium with priming technique was still slower than suxamethanium over onset time, it was still an optimal choice of rapid-sequence inducing under the condition of succinylcholine contraindication.^12^ Interestingly, ephedrine following rocuronium priming could improve the intubating condition,^13^ but ephedrine could not enhance the intubating condition following priming with atracurium.^14^

Reserchers^15^ found atracurium priming with 1/10 of intubating dose at 2-4 minutes ahead of intubating dose administration can accelerate onset time about 30-60s, and then tracheal intubation could be performed about 90s after the intubating dose was injected. However our results showed that atracurium priming can only speed up the onset time and shorten the intubating time for women, but not for men. Pharmacodynamics showed that gender was one of the affecting factors, and the onset time of atracurium in females was quicker than that in males.^16^ Age and gender could affect the pharmacodynamics of atracurium and the dose-response of atracurium was related to age and sex closely.^17^ The clearance of atracurium in males was greater than that in females, and the elimination in males was shorter than in females,but sex and age could not affect the volume of distribution of atracurium.^18^ Therefore women were more sensitive to neuromuscular blocking induced by atracurium than men.^16^ Our results showed the intubating time of female patients was shorter than that of male patients with atracurium priming. This illustrated that women were more sensitive to priming dose of atracurium than men. Studies have showed that the males needed more doses of atracurium to take effect than the females, and the duration of atracurium in males was shorter than that in females.^16^ We did not find significant differences between the males and the females without atracurium priming over intubating time, which might be explained that the intubating dose of atracurium was enough to take effect regardless of sensitivity discrimination. Moreover, for females, even after the intubating dose (0.5mg*kg^-1^) was infused, it still needed about 114s to reach the condition of tracheal intubation. These disagreements may ascribe to the different ethnicity to some extent, and the drug itself may also be one of the affecting factors.

We also observed the dizziness, diplopia and heavy eyelids, found incidences in females were higher than those in males. Other studies revealed that priming of atracurium led to heavy eyelids and double vision,^11^ and priming atracurium dose with 0.02mg/kg did not lead to orbicularis fade.^19^ Interestingly, one research thought females were more inclined to experience adverse effects to suxamethonium, and men were more likely to suffer an adverse effects to atracurium.^20^ However our research did not support this, the incidences of adverse effect to priming atracurium in females was higher than that in males. Atracurium priming could take effect on swallowing,^21^ and priming dose with 0.1 ED95 of atracurium was safe and did not enhance the risk of pre-curarization.^22^ Priming technique should be performed carefully, although most of these adverse effects were mild and no special treatments were needed. In this study we found that gender could affect the outcome of atracurium priming technique, and only female patients cloud benefit from atracurium priming. But what is the fundamental reason for this result? Maybe the differences of activity of plasma esterase between sexes^23^ is a possible reason. It is also possible that the threshold to muscular relaxant of different genders is different, or the amount or construction of N2-acetylcholine receptor cation channel is various between man and woman. It deserves further investigation to illustrate the mechanism.

## Authors Contribution

Liu Hui conceived the study, participated in its design, acquisition of data and drafting.

Gu Lianbing supervised the study and also participated in carrying out this study.

Zuo Yunxia took part in the design of the study and the writing of the manuscript.
